# A novel cyclic helix B peptide inhibits dendritic cell maturation during amelioration of acute kidney graft rejection through Jak-2/STAT3/SOCS1

**DOI:** 10.1038/cddis.2015.338

**Published:** 2015-11-26

**Authors:** C Yang, Y Zhang, J Wang, L Li, L Wang, M Hu, M Xu, Y Long, R Rong, T Zhu

**Affiliations:** 1 Department of Urology, Zhongshan Hospital, Fudan University, Shanghai, China; 2 Shanghai Key Laboratory of Organ Transplantation, Shanghai, China; 3 Department of Plastic Surgery, Zhongshan Hospital, Fudan University, Shanghai, China; 4 Biomedical Research Center, Zhongshan Hospital, Fudan University, Shanghai, China; 5 CAS Key Laboratory of Receptor Research, Shanghai Institute of Materia Medica, Chinese Academy of Sciences, Shanghai, China; 6 Department of Transfusion, Zhongshan Hospital, Fudan University, Shanghai, China

## Abstract

We recently synthesized a novel proteolysis-resistant cyclic helix B peptide (CHBP) that exhibits promising renoprotective effects. Dendritic cells (DCs) play an activation role in acute rejection (AR). Thus, the present study was designed to investigate the effects of CHBP on DCs in a rat renal transplantation model. The left kidney was harvested from male Lewis rats and then transplanted into male Wistar rats with or without CHBP treatment. Five successive treatment doses of CHBP after transplantation significantly ameliorated AR with lower histological injury, apoptosis and CD4^+^ and CD8^+^ T-cell infiltration in renal allografts. CHBP reduced IFN-*γ* and IL-1*β* levels but increased IL-4 and IL-10 levels in the serum. The number of mature DCs was significantly decreased in renal allografts treated with CHBP. In addition, incubating DCs with CHBP *in vitro* led to reduction in TNF-*α*, IFN-*γ*, IL-1*β* and IL-12 levels and increase of IL-10 expression at the protein level in the supernatant. Mechanistically, CHBP inhibited TLR activation-induced DC maturation by increasing SOCS1 expression through Jak-2/STAT3 signaling. In conclusion, CHBP suppresses renal allograft AR by inhibiting the maturation of DCs via Jak-2/STAT3/SOCS1 signaling, suggesting that CHBP may be an potential therapeutic drug for treating renal AR.

Renal transplantation has emerged as a viable therapeutic modality for the treatment of end-stage renal disease. However, there are multiple causes for the organ shortage crisis and the growth of the transplant wait list. Rejection is the major barrier to successful transplantation and acute rejection (AR) is considered to be an impediment to the short- and long-term survival of both renal allografts and recipients, and also a significant contributor to the escalating wait list due to the return of patients with a failed graft to the list.^1, 2^ AR of the donor organ is caused by antigen (Ag)-presenting cells (APCs) from the recipient presenting donor allopeptides loaded on self-major histocompatibility complex (MHC) molecules to T cells via the canonical pathway (the indirect pathway) as well as non-self, donor-intact MHC molecules through the direct pathway.^3^

For many years, AR has been considered to be a typical response of the adaptive immune system. However, recent investigations have revealed a critical role for the innate immune system as a pivotal trigger in adaptive immune responses.^4, 5, 6, 7^ During the recovery of an allograft from a donor, the process of recovery leads to the induction of stress in the allograft, including physical factors and ischemia reperfusion injury. These injuries caused by organ manipulation then induce the expression of damage-associated molecular patterns, such as heat-shock proteins, that are recognized by pattern recognition receptors (PRRs) localized on immune cells such as dendritic cells (DCs).^8^ Toll-like receptors (TLRs), which are important and typical PRRs, are activated by these danger signals and alert the DCs through the activation of transcription factors that encode the genes regulating inflammatory cells and mediators. Therefore, in the presence of an inflammatory milieu, DCs become mature, intercept Ags and present them to immunocompetent cells, activating adaptive immunity and favoring rejection.^9^

In addition to their well-known capacity to initiate immune responses, DCs are also increasingly considered to be mediators of transplant immune tolerance, including clonal deletion, the induction of T-cell anergy and the inhibition of memory T-cell responses.^10^ These properties have led to the use of immature DCs (imDCs) as a therapeutic strategy to induce solid organ transplant immune tolerance. In rodents, the infusion of donor- or recipient-derived imDCs can extensively prolong donor-specific allograft survival in association with the regulation of the host T-cell responses.^11, 12, 13^

Current therapy for AR mainly focus on immunosuppression, such as the application of glucocorticoids and antithymocyte globulin, which also accompanies with many side effects including hypertension and infection.^14, 15^ Therefore, it is urgent to develop an immnunoregulatory drug with low toxicity for AR treatment. Our group recently synthesized a novel proteolysis-resistant cyclic helix B peptide (CHBP), which has prominent anti-inflammatory characteristics.^16^ In our previous study, CHBP significantly ameliorated innate immunity-related inflammation in renal ischemia reperfusion injury in a murine model, in terms of decreased apoptosis, pro-inflammatory cytokines expression and complement activation.^16^ However, the influence of CHBP on either renal allograft rejection or innate immunocytes remains unknown. Therefore, we hypothesized that CHBP may have the ability to promote anti-rejection via the modulation of innate immunity, particularly pathogen-associated molecular patterns-induced activation and maturation of DCs. In the present study, we investigated and confirmed the effects of CHBP on anti-AR in a rat renal transplantation model and its ability to inhibit DC maturation *in vitro*. Moreover, we further clarified that the mechanism underlying the inhibition of TLR-induced DC maturation by CHBP is dependent on the activation of suppressor of cytokine signaling (SOCS) 1.

## Results

### CHBP treatment ameliorated AR and modulated apoptosis in renal allografts

**Figure 1:**
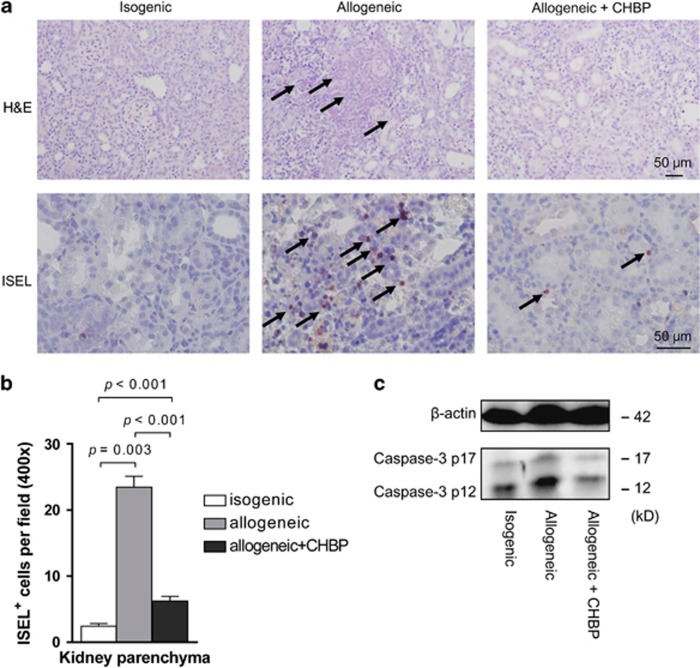
CHBP reduced AR and modulated apoptosis in kidney allografts. (**a**) Kidney tissue sections were stained with hematoxylin and eosin (H&E) and TUNEL assay. Scale bars, 50 *μ*m. (**b**) Semiquantitative analysis for apoptotic cells in the kidney parenchyma. (**c**) The expression of cleaved caspase-3 protein p12 and p17 was detected by western blotting. Results are mean±S.D. from three independent experiments. (**a** and **c**) Representative images from one experiment out of three are shown

Vehicle-treated renal allografts showed severe histological damage characterized by diffused heavy inflammatory cells infiltration with marked perivascular accentuation, widespread tubular injury with peritubular capillaritis and interstitial inflammation, and glomerulopathy on day 5 after transplantation. By contrast, the isogenic grafts exhibited no obvious damage. The CHBP-treated allografts showed less damage compared with the vehicle-treated group (Figure 1a).

The apoptotic cells in the transplant kidneys were examined by *in situ* end labeling (ISEL) of fragmented DNA. The apoptotic cells were mainly located in the tubular and interstitial areas (Figure 1a). Semiquantitative analysis revealed that the number of apoptotic cells in the kidney parenchyma was greatly increased in the allogeneic transplant kidneys compared with the isogenic control kidneys, but these cell numbers were reduced by CHBP treatment (Figure 1b). In addition, the expression of cleaved caspase-3 protein was significantly decreased in the CHBP-treated allogeneic transplant kidneys (Figure 1c). Taken together, these data demonstrated that CHBP treatment effectively attenuated the severity of AR and modulated apoptosis in renal allografts after transplantation.

### CHBP ameliorated systemic and local inflammation

**Figure 2:**
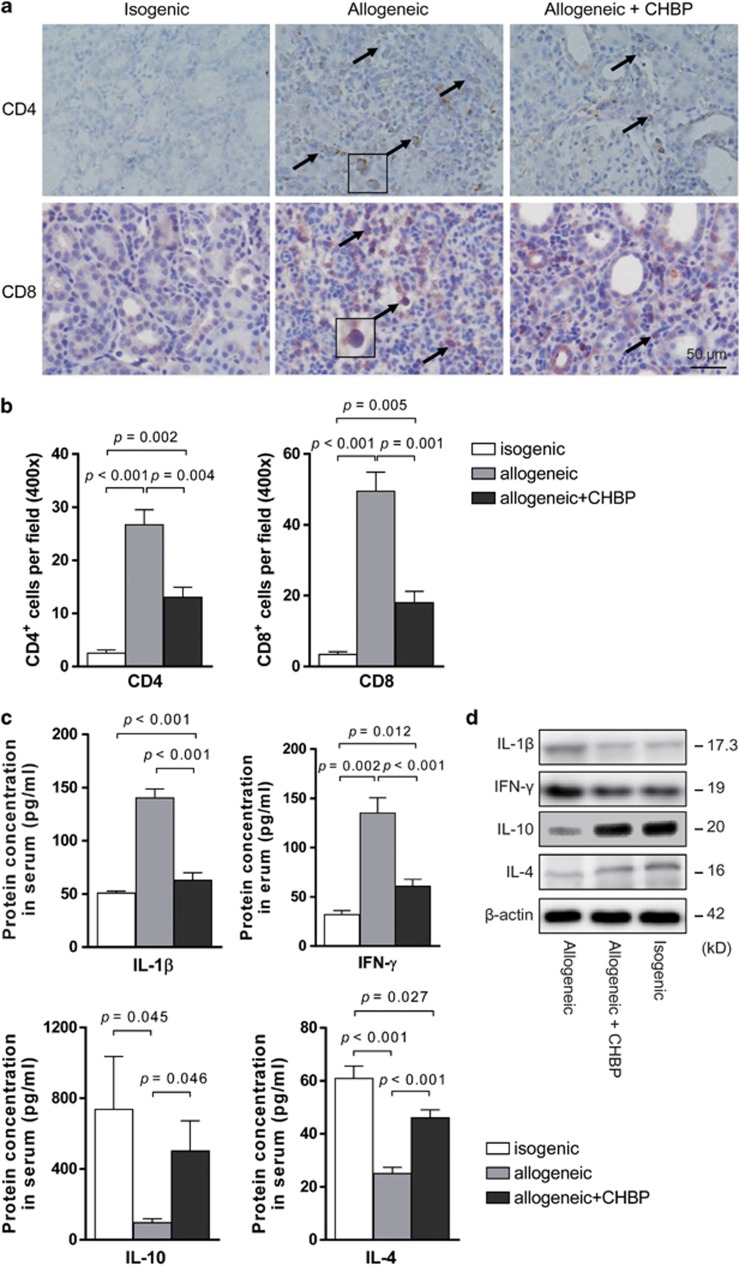
CHBP inhibited the infiltration of CD4^+^ and CD8^+^ T cells and ameliorated systemic and local inflammation. The expression of CD4 and CD8 in renal allografts was detected by immunohistochemical staining (**a**) and semiquantitative analysis for the number of CD4^+^ and CD8^+^ T cells was shown (**b**). Scale bar, 50 *μ*m. (**c**) Levels of IL-1*β*, IFN-*γ*, IL-4 and IL-10 in serum were detected by ELISA. (**d**) The expression of IL-1*β*, IFN-*γ*, IL-4 and IL-10 in kidneys was detected by western blotting. Results are mean±S.D. from three independent experiments. (**a** and **d**) Representative images from one experiment out of three are shown

To investigate the effect of CHBP on systemic and local inflammation, the infiltration of inflammatory cells was measured. As shown in Figure 2a and b, a massive infiltration of CD4^+^ and CD8^+^ T cells was evident in the interstitial area of vehicle-treated renal allografts. This infiltration was profoundly inhibited by CHBP, as shown by immunostaining on day 5, suggesting that CHBP inhibited the infiltration of T cells.

Then we tested the inflammatory cytokine expression in the peripheral blood by enzyme-linked immunosorbent assay (ELISA). IL-1*β* and IFN-*γ* levels were significantly decreased, whereas IL-4 and IL-10 levels were significantly increased by CHBP treatment (Figure 2c). In the transplant kidneys, the protein levels of IL-1*β*, IFN-*γ*, IL-4 and IL-10 exhibited a similar pattern to serum (Figure 2d). These results suggested that CHBP ameliorated systemic and local inflammation in renal allografts.

### CHBP suppressed mature DC location *in vivo*

**Figure 3:**
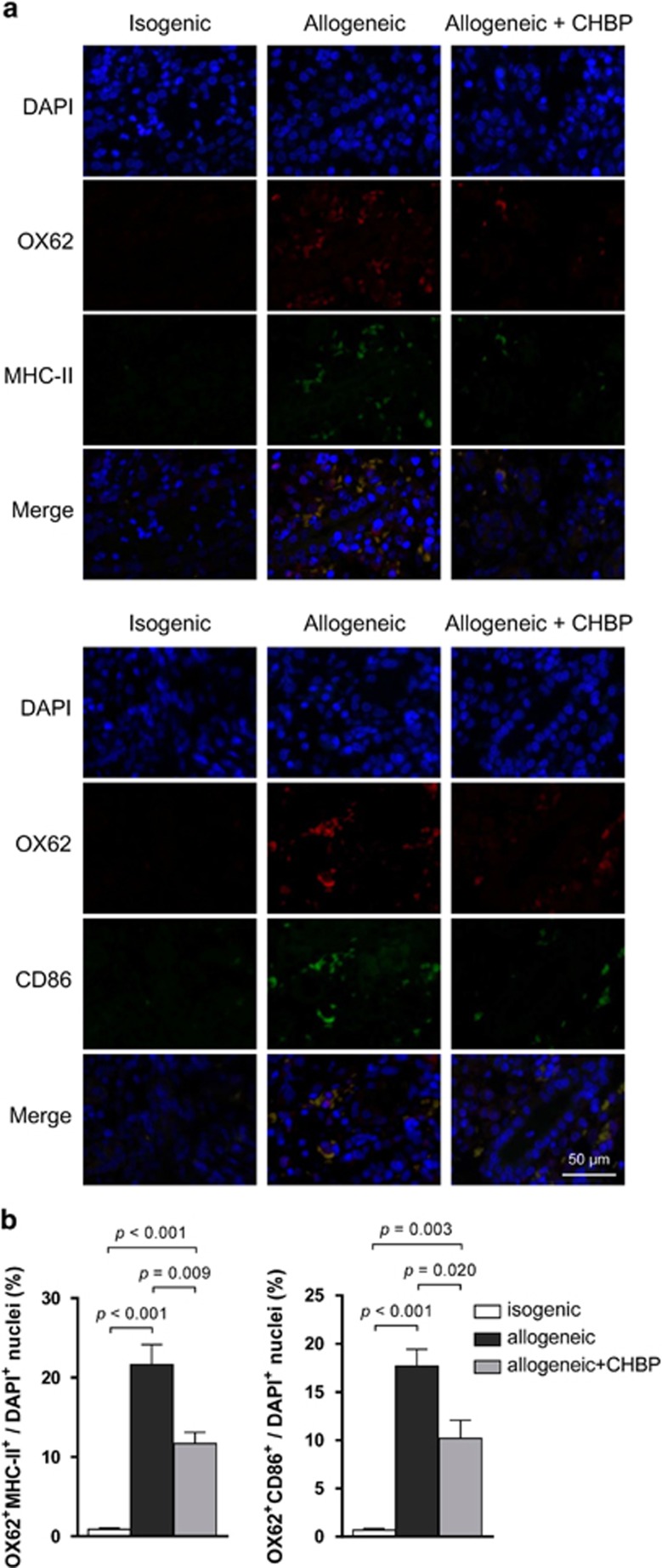
CHBP suppressed mDC location in the renal allograft. (**a**) The expression of OX62 combined with MHC-II or CD86 in renal allografts was detected by immunohistochemical staining. Scale bar, 50 *μ*m. (**b**) The ratios of double staining cells/DAPI^+^ cells. Results are mean±S.D. from three independent experiments. Representative images from one experiment out of three are shown

It has been reported that the majority of kidney-infiltrating effector T cells engage in stable contacts with DCs in the graft. Then the DC–T cell interactions amplify continuous stimulation and shape graft inflammation, resulting in graft injury.^17^ In this regard, we further examined DCs in the transplant kidneys through OX62, MHC-II and CD86 immunostaining (Figure 3a). Because OX62 is a specific marker of rat DCs and MHC-II and CD86 are vital for the Ag-presenting function of DCs, decreased numbers of OX62^+^ cells combined with MHC-II^+^ or CD86^+^ positive cells in the CHBP-treated renal allografts indicated that CHBP suppressed mature DC (mDC) location in the renal allografts (Figure 3b).

### CHBP inhibited bone marrow (BM)-derived DC maturation *in vitro*

**Figure 4:**
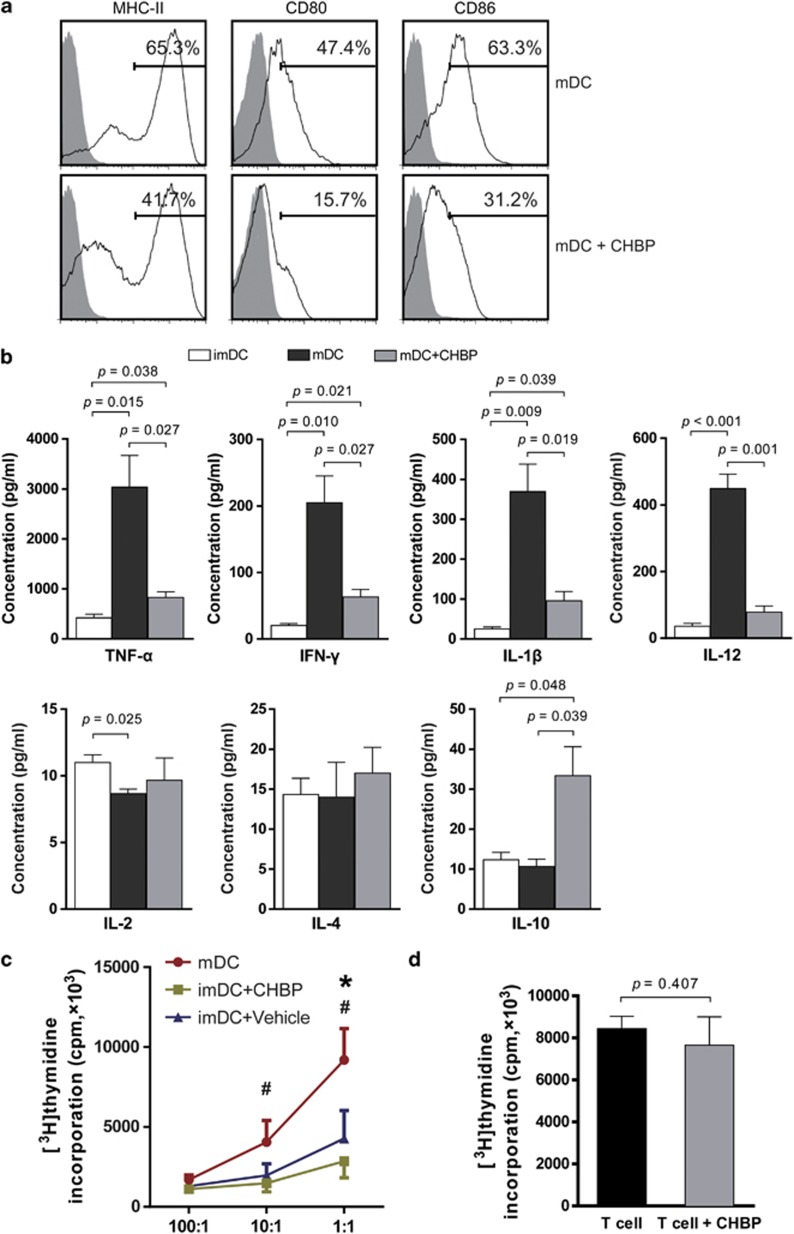
CHBP inhibited DC maturation *in vitro*. BM cells from Wistar rats were plated in 24-well plates with rGM-CSF (20 ng/ml) and rIL-4 (10 ng/ml) with or without CHBP (20 nmol/l). Cells were cultured for 8 days to induce imDCs and LPS was added in the culture system for another 24 h to induce mDCs. (**a**) The expression of MHC-II, CD80 and CD86 on mDCs was examined by flow cytometry. (**b**) Levels of TNF-*α*, IFN-*γ*, IL-1*β*, IL-2, IL-4, IL-12 and IL-10 in the supernatant were detected using ELISA. (**c**) The proliferation of T cells inhibited by DCs with or without CHBP incubation was evaluated by MLR (**d**) Direct effect of CHBP on T cells proliferation was examined by T cells proliferation assay. Results are mean±S.D. from three independent experiments. ^#^*P*<0.05 imDC+CHBP *versus* mDC, *P<0.05 imDC+CHBP *versus* imDC+Vehicle

To further investigate the role of CHBP in the maturation of DCs, we isolated Wistar rat BM-derived DCs for *in vitro* culture. Under lipopolysaccharide (LPS) stimulation, the expression of MHC-II, CD80 and CD86 on DCs was markedly reduced in the CHBP-treated group (Figure 4a). In addition, the incubation of BM-derived DCs with CHBP led to reduced levels of TNF-*α*, IFN-*γ*, IL-1*β* and IL-12 but increased IL-10 level in the supernatant (Figure 4b). To further compare the function of DCs with or without CHBP treatment, we performed mixed lymphocyte reaction (MLR) using gradient ratios between DCs and allogenic T cells. The proliferation of T cells was markedly inhibited by CHBP-incubated DCs compared with vehicle-treated DCs (Figure 4c). To exclude the direct effect on T-cell proliferation of CHBP, we further examined the T-cell proliferation with or without CHBP treatment and no significant difference was observed (Figure 4d). These results confirmed that CHBP played an immunosuppressive role in allogeneic immune responses by suppressing the maturation of BM-derived DCs.

### CHBP mediated Jak-2/STAT3/SOCS1 signaling to inhibit DC maturation

**Figure 5:**
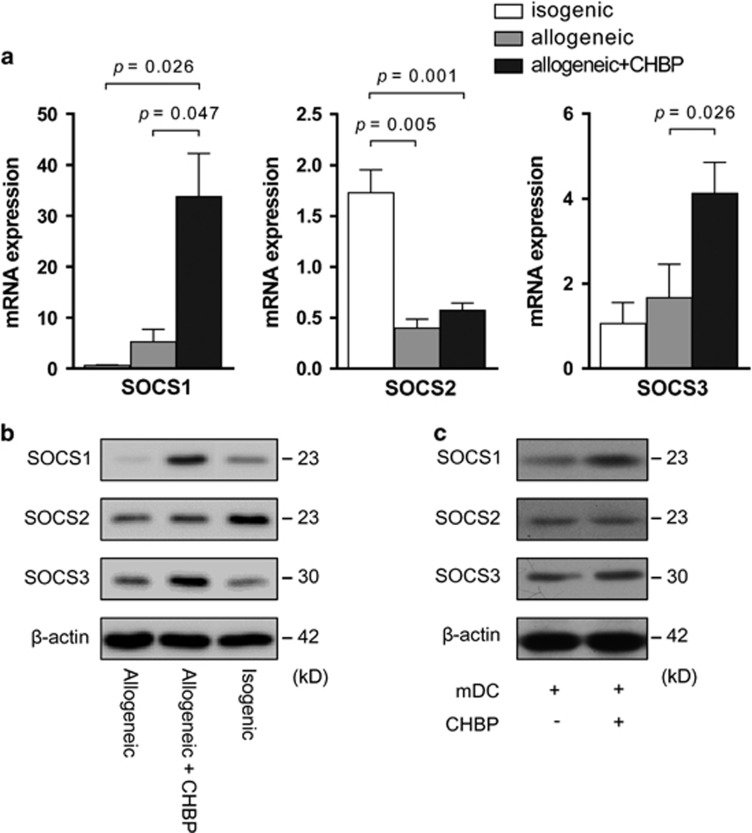
CHBP modulated SOCS expression in the transplant kidney *in vivo* and DCs *in vitro*. In the *in vivo* transplant model, the mRNA level and protein level of SOCS1, SOCS2 and SOCS3 were detected by RT-qPCR (**a**) and western blotting (**b**). (**c**) In the *in vitro* cell culture model, SOCS1, SOCS2 and SOCS3 protein levels in mDCs after LPS induction with or without CHBP (20 nmol/l) were detected by western blotting. Results are mean±S.D. from three independent experiments. (**b** and **c**) Representative images from one experiment out of three are shown

To investigate the mechanism through which CHBP inhibited DC maturation, we first examined the mRNA and protein levels of SOCS1, SOCS2 and SOCS3 in the transplant kidneys. Compared with the vehicle-treated group, both the mRNA and protein expression levels of SOCS1 and SOCS3 were significantly increased in the CHBP-treated group. Although the expression of SOCS2 was significantly increased in the isogenic grafts, there was still no significant difference between the two allogeneic groups with or without CHBP treatment (Figure 5a and b). Therefore, we further examined the protein levels of SOCS1, SOCS2 and SOCS3 in cultured DCs in the *in vitro* experiments. After incubation with CHBP, the SOCS1 protein levels but not SOCS2 or SOCS3 were significantly increased in the DCs (Figure 5c), implying the essential role of SOCS1 in the process of DC maturation.

**Figure 6:**
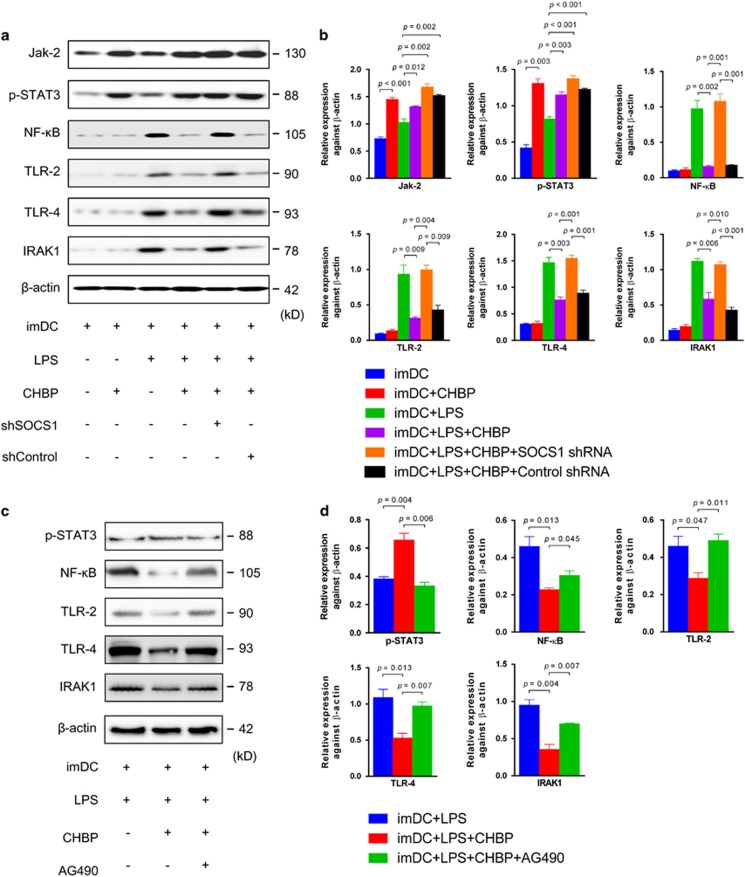
The inhibition of DC maturation by CHBP is mediated by Jak-2/STAT3/SOCS1 signaling. Cells were cultured for 8 days to induce imDCs and LPS was added in the culture system for another 24 h to induce mDCs. (**a**) The expression of Jak-2, p-STAT3, NF-*κ*B, TLR-2, TLR-4 and IRAK1 was examined by western blotting in BM-derived DC maturation model. (**b**) Semiquantitative analysis for western blotting was shown. (**c**) The expression of p-STAT3, NF-*κ*B, TLR-2, TLR-4 and IRAK1 was examined by western blotting in LPS-induced DCs maturation model. (**d**) Semiquantitative analysis for western blotting was shown. Results are mean±S.D. from three independent experiments. (**a** and **c**) Representative images from one experiment out of three are shown

Because LPS is a ligand of TLR-2 and TLR-4, the TLR-2/4 signaling pathway was studied by western blotting (Figure 6a). LPS activated TLR-2/4 signaling, as detected by increased TLR-2, TLR-4 and IRAK1 that is an adaptor protein downstream in the pathway. The activation of TLR-2/4 led to the increases of NF-κB, an important pro-inflammatory transcription factor in the TLR-2/4-mediated signal. After treatment with CHBP, the expression of TLR-2/4 was significantly downregulated. However, the effects of CHBP were reversed by SOCS1 knockdown using SOCS1 shRNA (Figure 6b). Moreover, downregulation of the expression of SOCS1 by SOCS1 shRNA was validated by qPCR and western blotting (Supplementary Figure S2). These results suggested that the CHBP-induced inhibition of DC maturation is mediated by the upregulation of SOCS1.

**Figure 7:**
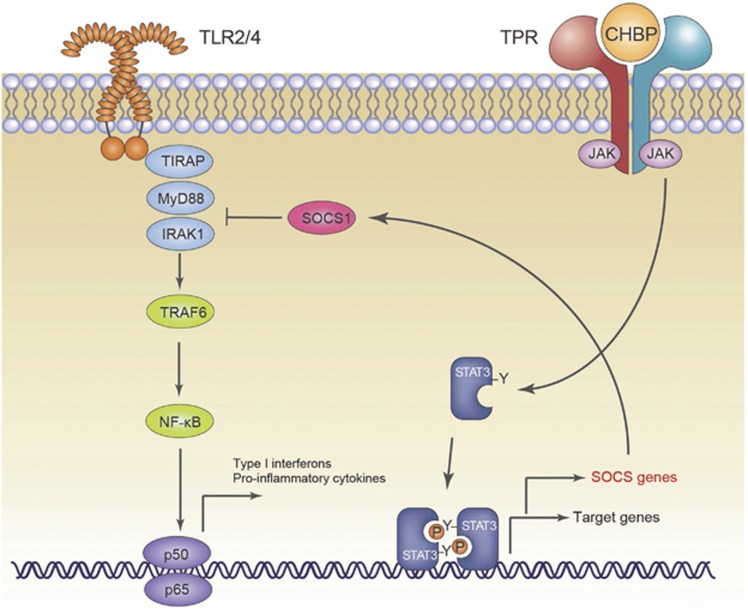
Schematic diagram demonstrating the signaling pathways involved in the inhibition of DC maturation by CHBP

To further determine the upstream signal regulation mechanism, we examined the Jak-2/STAT3 signal pathway, which is responsible for the activation of SOCS1. In the presence of CHBP, the expression of both Jak-2 and p-STAT3 in DCs was upregulated, regardless of SOCS1 shRNA treatment (Figure 6a and b). In addition, the suppressive effects of CHBP could be markedly inhibited by the Jak-2 inhibitor AG490, as shown in Figure 6c and d. CHBP also increased the expression of Jak-2 and p-STAT3 in the absence of LPS stimulation, indicating that it activates the Jak-2/STAT3 signal pathway. Therefore, the inhibition of BM-derived DC maturation by CHBP is mediated by the activation of Jak-2/STAT3/SOCS1 (Figure 7).

## Discussion

In this study, we demonstrate for the first time that the novel peptide CHBP ameliorates AR in a rat renal transplantation model. Mechanistically, CHBP inhibits the infiltration of T cells through suppressing DC maturation via the Jak-2/STAT3/SOCS1 signal pathway.

CHBP is a novel derivative of helix B surface peptide (HBSP).^18^ Based on our previous study, the linear peptide HBSP exerts strong renoprotective effects in renal ischemia reperfusion injury, in which model the innate immunity plays a predominant role.^19, 20^ With the induction of kidney parenchyma apoptosis, the pro-inflammatory cells such as macrophages and neutrophils are activated, and inflammation cascade subsequently aggravates kidney injury. However, the mechanisms of renal injury between ischemia reperfusion and AR are quite different. The fact that the innate immune system is necessary but not sufficient is well established. DC is a bridge connecting innate and adaptive immune system, and the allogeneic T-cell activation elicited by donor or recipient DC contributes directly to kidney AR. In this study, we found that the numbers of infiltrated CD4^+^ and CD8^+^ T cells were significantly decreased by treatment with CHBP, suggesting a failure in the full activation of anti-donor T-cell-mediated rejection. In a recent study, allogeneic grafts elicited persistent differentiation of monocytes into mDCs expressing IL-12 and stimulated T-cell proliferation and IFN-*γ* production. By contrast, the depletion of monocyte-lineage cells in the host at the time of transplantation significantly reduced T-cell infiltration.^21^ Therefore, our results indicated that CHBP also inhibited the adaptive immune response by inhibiting DC maturation.

OX62 is a specific marker of rat DCs.^22^ In the renal grafts, CHBP reduced the number of *in situ* DCs as well as their activation, as determined by the decreased levels of OX62, MHC-II and CD86 expression. MHC-II is an important Ag-presenting molecule of mDCs that are able to phagocytose Ags and present Ags to T cells via the peptide–MHC complex. CD80 and CD86 are co-stimulatory molecules as a second signal whose expression are upregulated when DCs are activated.^23^ Besides the decreased expression levels of CD80 and CD86, the MLR assay also showed that CHBP significantly inhibited the ability of DCs to activate T cells in the presence of alloreactive Ags. CHBP treatment, however, has no obvious effects on T cells directly. Therefore, these results demonstrated that CHBP played an immunosuppressive role in allogeneic immune responses by suppressing DC maturation. In addition, recent findings reveal that the infusion of imDCs ameliorates allograft rejection and prolongs graft survival.^24, 25^ In summary, manipulating DCs may be a prominent anti-rejection therapy for kidney transplantation in the future.

In this study, we also investigated the mechanism by which CHBP inhibits DC maturation. As a linear precursor peptide of CHBP, HBSP was found to ameliorate inflammation and apoptosis in the kidney by our group^19^ and other researchers.^26^ Both HBSP and CHBP derived from erythropoietin (EPO), previous study reported by Rocchetta *et al.*, revealed that EPO enhanced immunostimulatory properties of imDCs, beyond its erythropoietic and cytoprotective effects.^27^ Owing to the instability of HBSP, we designed and synthesized conformationally constrained CHBP with significantly increased resistance to proteolytic degradation and improved renoprotective potency based on the amino acid sequence of HBSP.^16^ CHBP and HBSP share the same receptor, named tissue protective receptor (TPR), which is a heterodimer receptor composed of EPO receptor (EPOR) and *β*cR.^28^ Jak-2 is a downstream adaptor protein of TPR signaling that subsequently activates STAT3.^28^ Interestingly, EPO stimulation of the BM-DCs leads to Tyr phosphorylation of STAT3, suggesting STAT3 may play a more important role in EPOR signaling in BM-DCs.^29^ In the present study, we provide the first demonstration that CHBP activates the Jak-2/STAT3 signaling pathway in imDCs.

Although the activation of Jak-2/STAT3 does not directly influence the TLR-2/4-mediated signaling pathway, STAT3 induces the expression of proteins in the SOCS family, such as SOCS1, SOCS2 and SOCS3. SOCS proteins are key regulators of cytokine signaling and are important for maintaining balance in the immune system. These proteins are thought to participate in negative feedback loops in cytokine signaling.^30^ We found that the mRNA expression of SOCS1 and SOCS3 in the transplant kidney was significantly increased in the CHBP group compared with the allogeneic control group. Because SOCSs negatively regulate TLR signaling.^31^ We further examined the expression of SOCS1, SOCS2 and SOCS3 at the protein level in the CHBP-treated mDCs. Interestingly, only SOCS1 was significantly increased. Thus, the results suggest that the Jak-2/STAT3/SOCS1 pathway is involved in the negative regulation of TLR-2/4 signaling by treatment with CHBP.

Recent studies have revealed that silencing of the *socs1* gene in DCs enhances DC maturation and thus increases antitumor immunity.^32^ Moreover, in the SOCS1^−/−^ mouse, the developmental process for T cells in the thymus is disordered, and the peripheral T cells exhibit abnormal activation accompanied by Th2 cells in a disturbed state. SOCS1^−/−^ macrophages produce large amounts of inflammatory cytokines in response to lipopolysaccharide.^33^ These studies indicate that SOCS1 is an important regulator of DC maturation. In the present study, TLR-2/4 signaling in imDCs was rescued by SOCS1 shRNA even after treatment with CHBP. Thus, our results prove that SOCS1 is the key regulator involved in the CHBP-mediated inhibition of DC maturation.

In this study, we transplanted renal allografts orthotopically into unilaterally nephrectomized recipient rats. There are some advantages to transplanting renal allografts into unilaterally nephrectomized recipient rats. First, the unilaterally nephrectomized transplant model is an established model to investigate allogeneic immune responses after transplantation, including allogeneic immunocytes infiltration and histological damages, although renal function in terms of serum creatinine and blood urine nitrogen could not be unchanged (Supplementary Figure S1). Second, unilaterally nephrectomized recipients exhibited better general conditions than bilaterally nephrectomized recipients after transplantation, particularly in terms of recipient survival. However, this study had some limitations. First, the impact of CHBP on other APCs, such as macrophages and B cells or adaptive immunocytes, still requires further investigation. Second, the different doses of CHBP should also be tested in an *in vivo* model for further preclinical research.

In conclusion, this study demonstrates that the novel peptide CHBP effectively protects renal allografts against AR. The anti-AR effects of CHBP are mediated through the inhibition of DC maturation via the Jak-2/STAT3/SOCS1 signaling pathway. These results suggest that CHBP may be an effective therapeutic drug for renal allograft rejection and provide invaluable data for potential clinical translation in the future.

## Materials and Methods

### Rat acute renal allograft rejection model

An orthotopic renal transplantation model was performed using male Lewis rats (Weitonglihua Company, Beijing, China) and male Wistar rats (SLAC Laboratory Animal Co., Ltd., Shanghai, China), weighing 180–200 g. The MHC is incompatible between Lewis rats and Wistar rats. To establish the AR model, the donor kidney removed from a Wistar rat was transplanted orthotopically into a unilaterally nephrectomized Lewis recipient with end-to-end anastomosis of the renal artery, vein and ureter. All of the rats were bred in an experimental SPF-grade animal room. No immunosuppressive agents were used. The recipients were divided into three groups (n=5 for each group): (1) isogenic group, from Lewis to Lewis; (2) allogeneic group, from Wistar to Lewis with saline treatment; and (3) CHBP group, from Wistar to Lewis with CHBP treatment. The rats in the CHBP-treated group were administered 24 nmol/kg/day CHBP i.p. once per day after transplantation from day 0 to day 5, and the same dose of 0.9% saline as a vehicle was injected into the rats in the allogeneic group. All of the animal experiments were performed according to the guidelines of the Care and Use of Laboratory Animals of the Laboratory Animal Ethical Commission of Fudan University.

### Histological analysis of allograft rejection

Kidney tissue sections were stained with hematoxylin and eosin. Injured tubules were identified with one or more of the following characteristics: tubular atrophy, loss of brush border, loss of nuclei, basement membrane disruption and detachment of epithelial cells. More than 600 tubules were examined in each animal using 15 high-power fields in the renal cortex, and the results were expressed as percentages of injured tubules. The Banff interstitial inflammation score on a scale of 0 to 3 was used based on the percentage of cortical tissue affected by parenchymal inflammation (0, <10% 1+, 10–25% 2+, 26–50% 3+, >50%). Twenty high-power fields in the renal cortex were scored for each animal, and scoring was performed on blinded slides.

### ISEL apoptotic cells

ISEL apoptotic cells were detected using a TUNEL Apoptosis Detection Kit (Millipore, Billerica, MA, USA) as described previously.^16^

### Immunohistochemistry

Immunohistochemical staining of CD4, CD8, OX62, MHC-II and CD86 (Abcam, Cambridge, UK) was performed on paraffin-embedded or frozen sections using a DAKO ChemMate EnVision Detection Kit (DAKO, Carpinteria, CA, USA) as described previously.^19, 34, 35, 36^ The fluorescence intensity of OX62 and MHC-II or CD86 coexpression areas was quantitated using ImageJ software (National Institutes of Health, Bethesda, MD, USA).

### ELISA

The cytokines IL-1*β*, IL-2, IL-4, IL-10, IL-12, TNF-*α* and IFN-*γ* were assayed in duplicate using a sandwich ELISA (R&D Systems, Minneapolis, MN, USA). The sample preparation and procedure were performed according to the manufacturer's instructions.

### Western blotting

Twenty micrograms of proteins from the kidneys or DC homogenates were separated on 15 or 10% (wt/vol) polyacrylamide denaturing gels and electro-blotted onto polyvinylidene fluoride membranes. The primary antibodies used were anti-caspase-3 (1 : 500; Santa Cruz Biotechnology, Santa Cruz, CA, USA), IL-1*β* (1 : 1000; Cell Signaling Technology, Danvers, MA, USA), IFN-*γ* (1 : 1000; Cell Signaling Technology), IL-4 (1 : 1000; Cell Signaling Technology), IL-10 (1 : 1000; Cell Signaling Technology), SOCS1-3 (1 : 1000; Cell Signaling Technology), Jak-2 (1 : 1000; Cell Signaling Technology), phospho-STAT3 (1 : 1000; Cell Signaling Technology), NF-κB (1 : 1000; Cell Signaling Technology), TLR-2 (1 : 1000; Cell Signaling Technology), TLR-4 (1 : 1000; Cell Signaling Technology) and IRAK1 (1 : 1000; Cell Signaling Technology). The semiquantitative analysis (AlphaView Software 3.3; ProteinSimple, San Jose, CA, USA) results were expressed as the optical volume densities (OD x mm^2^) normalized to *β*-actin (1 : 10 000 dilution; Abcam).

### Real-time quantitative PCR (qPCR)

The mRNA expression levels of SOCS1, SOCS2 and SOCS3 were measured by real-time qPCR as described previously.^16^ The sequences of the primers are listed in Supplementary Table S1.

### shRNA knockdown assay

The shRNA sequence for SOCS1 (GGAACTGCTTCTTCGCGCTCA) and the negative control sequence (TTCTCCGAACGTGTCACGT) were cloned into the lentiviral pLKO.1 vector. Viral production was performed in 293 T cells via the cotransfection of the lentiviral vector pLKO.1 and packaging plasmids. The cells were incubated overnight at 37 °C with 5% CO_2_. The supernatants were collected at 48 h post-transfection, and the virus stock solution was used to infect marrow cells. The protein levels of SOCS1 were detected by western blotting.

### Flow cytometry analysis

Monoclonal antibodies with the following specificities were obtained from BD Biosciences (San Diego, CA, USA): CD103 (OX62), MHC-II (HIS19), CD80 (3H5) and CD86 (24 F). Multiple-color flow cytometric analysis was performed using a FACS Aria instrument (BD Biosciences).

### BM preparation and DC induction

Maturation of rat DCs was induced as previously described.^37^ Briefly, femurs and tibiae of female Wistar rats were removed and purified from the surrounding muscle tissue. Intact bones were left in 70% ethanol for 2–5 min for disinfection and washed with PBS. Then both ends were cut with scissors and the marrow flushed with RPMI 1640 using a syringe with a 0.45- mm diameter needle. The marrow was suspended, passed through a nylon mesh to remove small pieces of bone and debris, and red cells were lysed.

After washing, 10^6^ cells were plated in 24-well plates in 1 ml medium that was further supplemented with rGM-CSF (20 ng/ml) and rIL-4 (10 ng/ml) (R&D Systems) with or without CHBP (20 nmol/l). Cultures were fed every second day; 75% of the medium was aspirated and replaced with fresh medium containing cytokines and/or CHBP for 8 days to induce imDCs. Then, cells were centrifuged, dispersed in fresh media without cytokines and subcultured at 8 × 10^5^/ml in 100 mm dishes for 24 h with LPS stimulation (100 ng/ml). After overnight culture, large numbers of typical floating mDCs, covered with large veiled processes, were harvested.

### MLR

Mononuclear cells (MNCs) of spleens from Lewis rats were obtained by Ficoll density gradient centrifugation. CD4^+^ T cells were magnetically purified from MNCs according to the manufacturer's recommendations (Miltenyi Biotec, Auburn, CA, USA). The purity of sorted cells in this study was consistently more than 95%. BM-derived DCs from Wistar rats with or without CHBP treatment were lethally irradiated (30 Gy). The DCs were then cultured in graded doses with CD4^+^ T cells (3 × 10^5^ cells/well) isolated from Lewis rats in RPMI 1640 medium for 5 days. [^3^H]Thymidine (1 *μ*Ci/well; Shanghai Institute of Applied Physics, Chinese Academy of Sciences, Shanghai, China) was added 18 h before the end of the culture period. The cells were then harvested onto glass fiber mats for the measurement of [^3^H]thymidine incorporation.

### T-cell proliferation

CD4^+^ T cells were magnetically purified from spleens of Wistar rats according to the manufacturer's recommendations (Miltenyi Biotec). Then, CD4^+^ T cells (2 × 10^5^ cells/well) in triplicate 96-well, round-bottom culture plates were stimulated with plated-coated anti-CD3 antibody (5 *μ*g/ml) and soluble anti-CD28 antibody (1 *μ*g/ml) (both from eBioscience, San Diego, CA, USA) in RPMI 1640 medium with or without CHBP (20 nmol/l) for 72 h. [^3^H]Thymidine (1 *μ*Ci/well) was added 18 h before the end of the culture period. The cells were then harvested onto glass fiber mats for the measurement of [^3^H]thymidine incorporation.

### Statistical analysis

Results are expressed as the means±standard deviation (S.D.). Normality tests were carried out, and statistical analysis of the data was performed through one-way ANOVA using SPSS 18.0 software (SPSS Inc., Armonk, NY, USA). *P*<0.05 was considered statistically significant.

## Abbreviations

Ag: antigen
APC: Ag-presenting cell
AR: acute rejection
BM: bone marrow
CHBP: cyclic helix B peptide
DC: dendritic cell
ELISA: enzyme-linked immunosorbent assay
EPO: erythropoietin
HBSP: helix B surface peptide
imDC: immature DC
ISEL: *in situ* end labeling
LPS: lipopolysaccharide
mDC: mature DC
MHC: major histocompatibility complex
MLR: mixed lymphocyte reaction
MNC: mononuclear cells
PRR: pattern recognition receptors
qPCR: quantitative PCR
S.D.: standard deviation
SOCS: suppressor of cytokine signaling
TLR: Toll-like receptor
TPR: tissue protective receptor
