# Supplementary figures and images for: A novel cyclic helix B peptide inhibits dendritic cell maturation during amelioration of acute kidney graft rejection through Jak-2/STAT3/SOCS1
Source: Cell Death Dis. 2015 Nov 26;6(11):e1993–. doi: 10.1038/cddis.2015.338 (PMC4670942; doi:10.1038/cddis.2015.338)

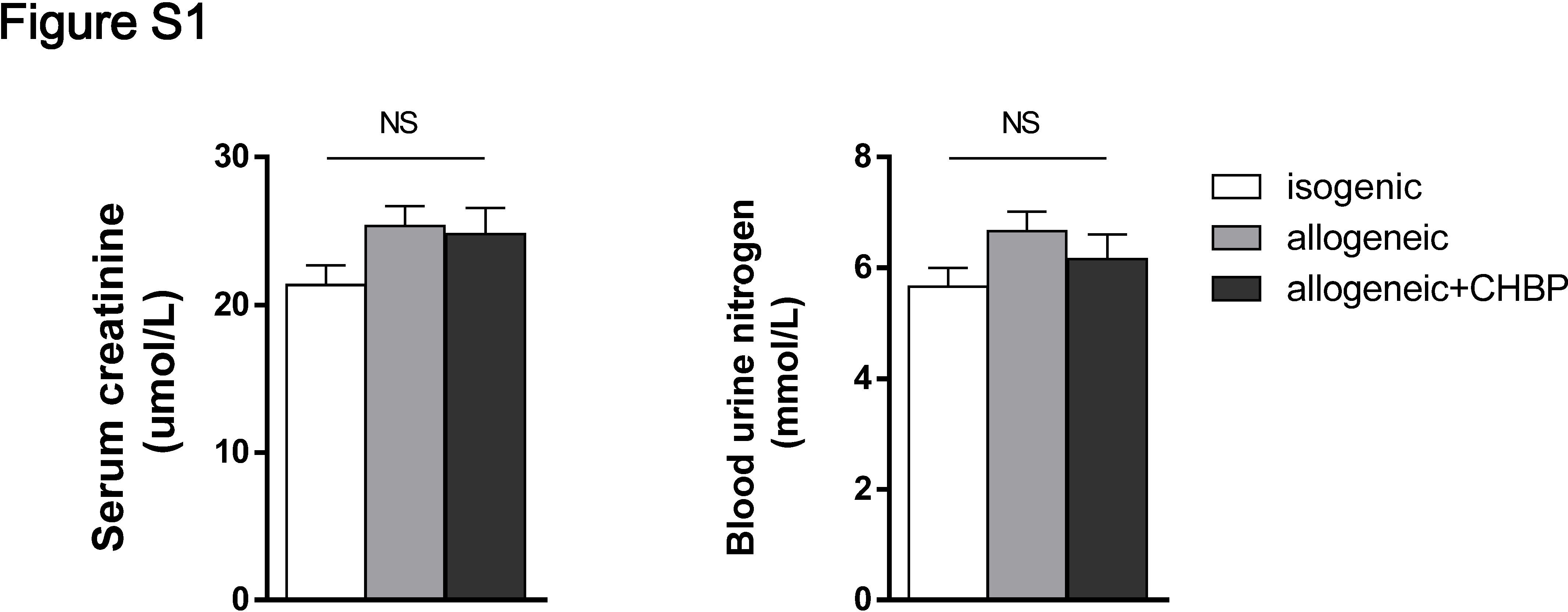

Supplement: Supplementary Figure S1 [file cddis2015338x1.tif]

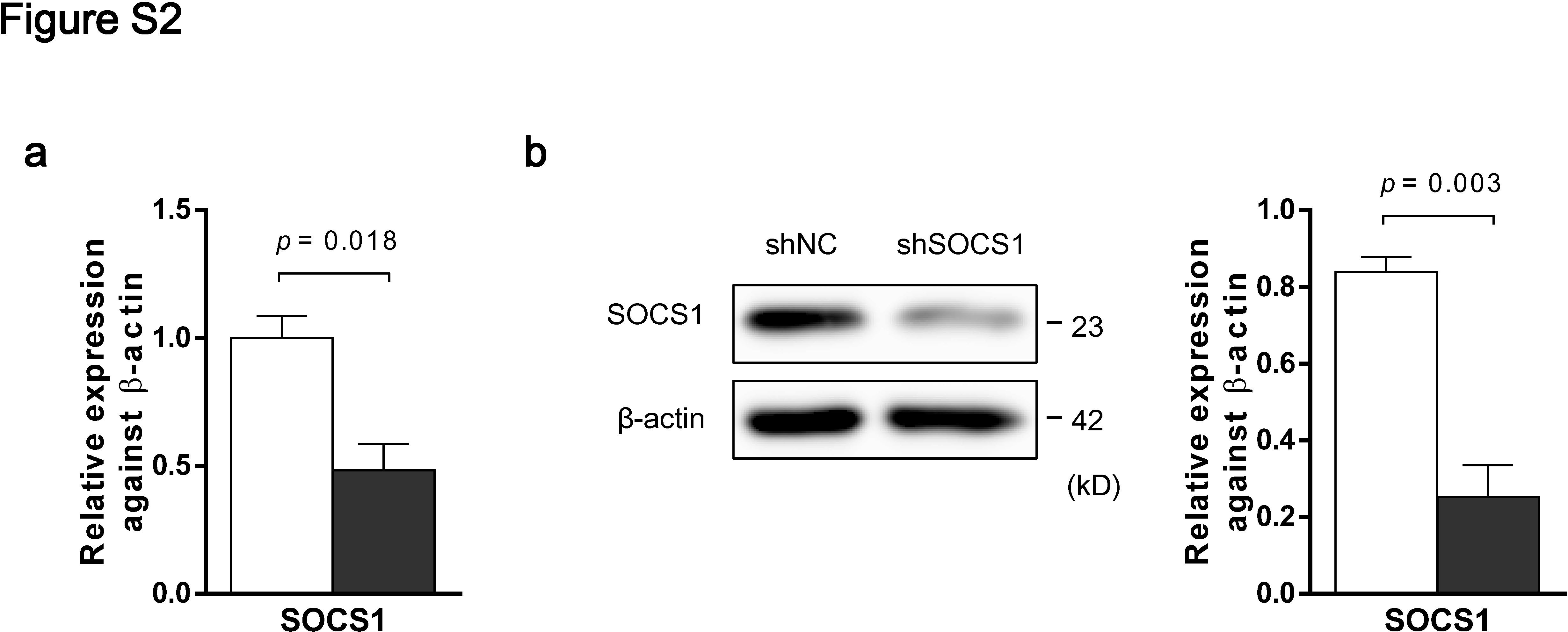

Supplement: Supplementary Figure S2 [file cddis2015338x2.tif]
