# Supplementary material for: A novel cyclic helix B peptide inhibits dendritic cell maturation during amelioration of acute kidney graft rejection through Jak-2/STAT3/SOCS1
Source: Cell Death Dis. 2015 Nov 26;6(11):e1993–. doi: 10.1038/cddis.2015.338 (PMC4670942; doi:10.1038/cddis.2015.338)
Supplement: Supplementary Figure Legends [file cddis2015338x3.doc]

**Figure S1**: **Renal function.**

Levels of the serum creatinine (a) and blood urine nitrogen (b) post transplantation were shown. Results are mean ± S.D. from three independent experiments.

**Figure S2**: **Validation of SOCS1 shRNA.**

The SOCS1 (a) mRNA and (b) protein expression of DCs with or without SOCS1 shRNA transfection were examined. Results are mean ± S.D. from three independent experiments.
