# Supplementary material for: A novel cyclic helix B peptide inhibits dendritic cell maturation during amelioration of acute kidney graft rejection through Jak-2/STAT3/SOCS1
Source: Cell Death Dis. 2015 Nov 26;6(11):e1993–. doi: 10.1038/cddis.2015.338 (PMC4670942; doi:10.1038/cddis.2015.338)
Supplement: Supplementary Table S1 [file cddis2015338x4.doc]

**Table S1 Primer sequences**

| Gene name |  | Primer sequence |
| --- | --- | --- |
| GAPDH | Sense | TGAGGCCGGTGCTGAGTATGT |
|  | Antisense | CAGTCTTCTGGGTGGCAGTGAT |
| NF-κB | Sense | AGCTCCTGTCCCAGTTCTAGC |
|  | Antisense | ACTCCTGGGTCTGTGTTGTTG |
| MyD88 | Sense | CGACGCCTTCATCTGCTACTGC |
|  | Antisense | CCACCACCATGCGACGACAC |
| SOCS1 | Sense | TGATTACCGGCGCATCAC |
|  | Antisense | GAAGCCATCTTCACGCTGAG |
| SOCS2 | Sense | GACGGGAAATTCAGATTGGA |
|  | Antisense | AATGCTGAGTCGGCAGAAGT |
| SOCS3 | Sense | CTGGACCCATTCGGGAGTTC |
|  | Antisense | AACTGGGAGCTACCGACCATTG |
